# A Bird’s-Eye Perspective: An Unusual Case of Very Late-Onset Schizophrenia-Like Psychosis With Visual Hallucinations Included in Its Manifestations Versus the Dementia Prodrome

**DOI:** 10.7759/cureus.57510

**Published:** 2024-04-03

**Authors:** Apurva Bezalwar, Pradeep S Patil, Ishaan Gautam, Namita Sahu

**Affiliations:** 1 Department of Psychiatry, Datta Meghe Institute of Medical Sciences, Wardha, IND; 2 Department of Psychiatry, Jawaharlal Nehru Medical College, Datta Meghe Institute of Higher Education and Research, Wardha, IND

**Keywords:** schizophrenia-like, cognitive, dementia, psychosis, hallucinations

## Abstract

Very late-onset schizophrenia-like psychosis (VLOSLP) is still a paradox; certain characteristics such as episodic progression of psychosis including delusions and hallucinations involving various modalities, as well as the absence of negative symptoms, are strongly predictive of VLOSLP. We describe an interesting case of a 61-year-old male who presented with a second episode of psychosis along with mild to moderate cognitive impairment like having difficulty in buttoning for over eight months at our tertiary care hospital. Previously, during the first episode, he was treated by a private practitioner; adequate doses for an adequate duration of two atypical antipsychotics were given; and up to 25% global improvement was reported by the caregiver. During the current episode, he experienced delusions, in which he had a conviction that a "WIFI" was capable of "thought-making" functions. During the past four months, his delusions exacerbated and were accompanied by hallucinations of other modalities, like visual and kinesthetic hallucinations, which profoundly impacted his daily life. He used to hear voices. While listening to the voices, he also experienced voices coming out of his mouth. All these were experienced by him in clear consciousness daily for a few hours. All plausible medical causes of late-onset psychosis, such as neuroinflammatory/immunological disorders, were ruled out. Neuroimaging revealed T2-weighted image (T2WI)/fluid-attenuated inversion recovery (FLAIR) hyperintensity in bilateral subcortical and periventricular deep white matter, suggestive of small vessel ischemic changes in the brain.

The diagnosis of VLOSLP is completely rationalized by evidence-based medicine. Hence, the role of cerebrovascular risk factors, as well as age-related neurobiological processes, in the pathogenesis of VLOSLP is discussed. Future research ought to emphasize identifying a particular biomarker that would be highly predictive for accurately diagnosing VLOSLP and giving it an identity to separate it from various overlapping clinical conditions such as dementia with Lewy bodies (DLB) and other types of dementia with psychosis so that the patient can be given specific treatment.

## Introduction

The two most notable distinctions between the presentation of individuals with very late-onset schizophrenia-like psychosis (VLOSLP) and those with early-onset schizophrenia (EOS) are the low frequencies of formal thought disorder and predominantly negative symptoms in the former group. Also, delusions belonging to the late-onset schizophrenia (LOS) and VLOSLP groups are more likely persecutory in nature. Moreover, there is an increased prevalence of visual, tactile, and auditory hallucinations, or the third person running commentary. Although not pathognomic, partition delusions are more common in the older cohort. The diagnosis of psychosis that develops late in life can be difficult because of the variety of neurobiological changes that occur as the brain ages as well as the emergence of neurological and physical conditions. Uncertainty surrounds the schemas and pathogenesis of VLOSLP. Since there are no particular biomarkers, the diagnosis uses clinical evidence, highlighting the significance of a detailed history and diagnostic evaluation.

Although the VLOSLP criteria state that psychosis should not be attributed to affective disorders or focal or structural brain abnormalities, it is unknown whether psychotic symptoms in VLOSLP are related to neurodegenerative diseases. Further research is needed to determine if there is a potential link between psychotic symptoms in VLOSLP and neurodegenerative diseases. Understanding this connection could provide valuable insights into the underlying mechanisms of VLOSLP and potentially lead to improved diagnostic and treatment approaches [1].

The community prevalence of VLOSLP ranges from 0.1% to 0.5%. However, with each five-year increase in age, the incidence of VLOSLP rises by 11% annually [2]. Therefore, differentiating between neurodegeneration and VLOSLP may be difficult for the elderly. Nonetheless, it is quite useful in terms of early treatment options.

## Case presentation

A 61-year-old retired male presented with episodic progression of psychosis along with mild to moderate cognitive impairment for over eight months at our tertiary care hospital. Previously, he was receiving regular treatment from a private psychiatrist. Although adequate doses for an adequate duration of two atypical antipsychotics were given, up to 25% global improvement was reported by the caregiver. Therefore, he was treated in inpatient care at our hospital.

During his hospital stay, he repeatedly complained that he experienced delusions, in which he had a conviction that a "WIFI" was capable of "thought-making" functions. These involved the surveillance, extraction, and replacement of his thoughts with others. During the past four months, his delusions exacerbated and were accompanied by hallucinations of other modalities, like visual and kinesthetic hallucinations. He was able to see shapes or people that weren't there, and these were accompanied by a sensation of touch at times, which profoundly impacted his daily life. He further added that there were “voices” of unfamiliar men and women, coming from a nearby distance, who discussed his daily activities and commented on him in an abusive or threatening manner, especially when alone. His psychopathology was characterized by thought alienation and somatic passivity. While listening to the voices, he also experienced voices coming out of his mouth, uttering abusive words that were not under his control. He would experience these in clear consciousness every day for a few hours.

No negative symptoms were observed. Previous substance history, psychiatric history, head injury, and medical comorbidities were ruled out during the initial assessment. On extensive evaluation, it was reported that he had difficulty buttoning and unbuttoning his shirt, dropping off his granddaughter at school, and signing bank checks over the past eight months.

**Figure 1:**
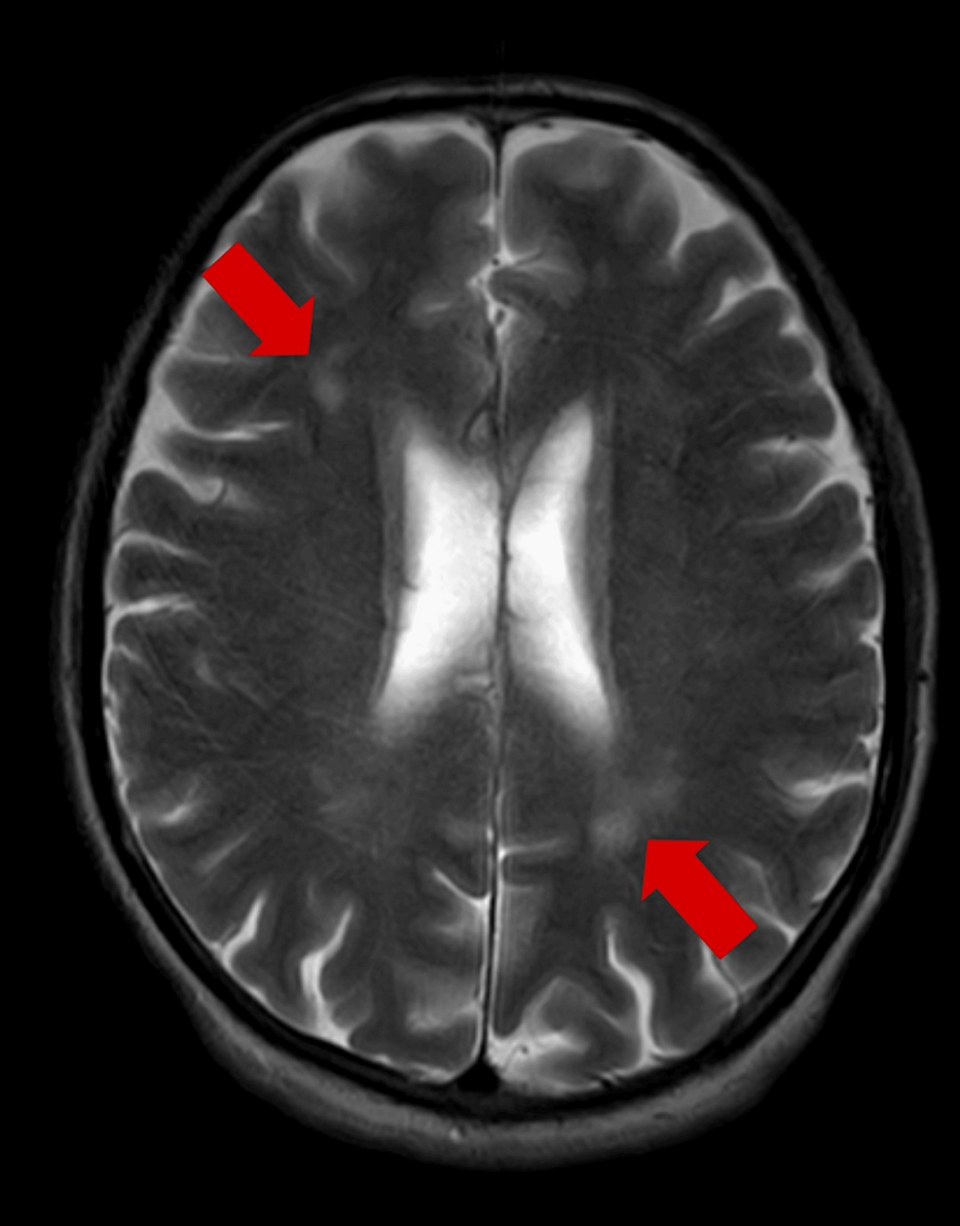
Noncontrast MRI brain image: T2 axial section showing hyperintensity in the bilateral subcortical and periventricular deep white matter The patient's brain volume is good and no cortical atrophy is observed in terms of dementia.

**Figure 2:**
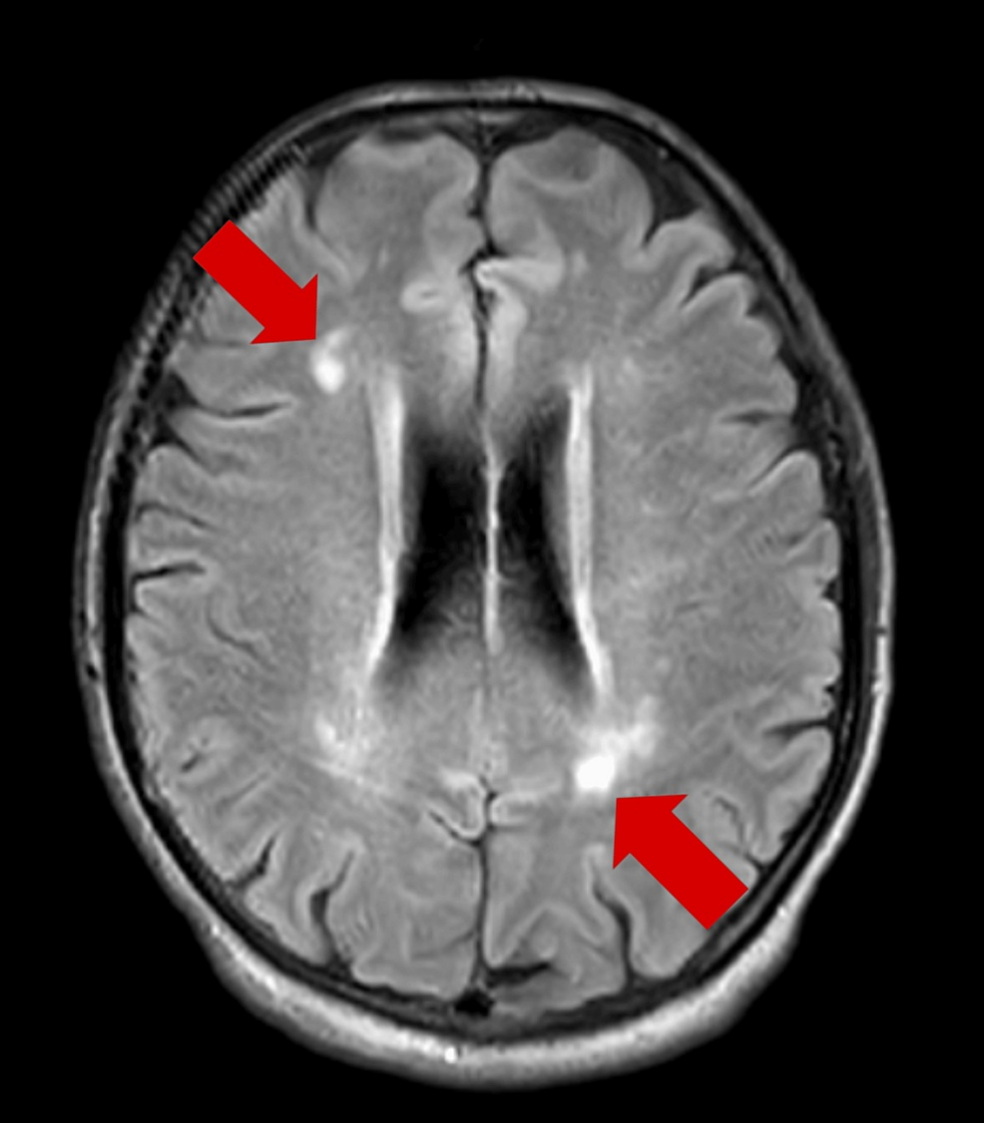
Noncontrast MRI brain image: Fluid-attenuated inversion recovery (FLAIR) axial section showing hyperintensity in bilateral subcortical and periventricular deep white matter Furthermore, there is no evidence of cortical atrophy in relation to dementia, and the patient's brain volume is good.

Blood investigations and a biochemistry panel revealed normal thyroid function and vitamin B9, B12, and vitamin D levels. All serological tests like hepatitis B and C, human immunodeficiency virus (HIV), venereal disease research laboratory (VDRL), and anti-thyroid peroxidase (anti-TPO) antibodies were negative. Electroencephalography findings were normal. Magnetic resonance imaging (MRI) revealed T2-weighted image (T2WI)/fluid-attenuated inversion recovery (FLAIR) hyperintensity in the bilateral subcortical and periventricular deep white matter, suggestive of small vessel ischemic changes in the brain. The noncontrast MRI brain images of the patient are shown in Figure 1 and Figure 2.

Higher mental function examination revealed delayed recent and immediate recall, ill-sustained attention, impaired abstraction, and impaired visuospatial faculty. The Mini-Mental State Examination (MMSE) score was 17/30, while the Addenbrooke's Cognitive Examination (ACE III) score was 61/100. 

Significant improvement in symptoms was perceived after resuming olanzapine 10 mg. Further, it was increased to 20 mg. As the psychotic symptoms reduced, cognitive deficits got better. The MMSE score and ACE III score improved to 24 and 86, respectively. On discharge, the psychotic symptoms decreased significantly. He was able to function adequately, like buttoning-unbuttoning his shirt, dropping his granddaughter to school, and signing checks. He was able to carry out even instrumental activities of daily living without any assistance. Diagnosis of VLOSLP was made according to the International Classification of Diseases 11th Revision (ICD 11) and the Diagnostic and Statistical Manual of Mental Disorders, Fifth Edition, Text Revision (DSM-5-TR).

## Discussion

The differential diagnosis is quite challenging. Studies show that hallucinations in VLOSLP are primarily visual or auditory and are mostly associated with paranoid delusions. However, animal and human visual hallucinations uniquely predicted the diagnosis of dementia with Lewy bodies (DLB). Delusions and hallucinations of other modalities without any associated negative symptoms are common clinical presentations of a VLOSLP [3]. VLOSLP is often associated with cognitive impairments, particularly in learning and abstract thinking. According to studies, hallucinations of human voices predicted a diagnosis of VLOSLP [4].

It is dubious whether his cognitive impairment was caused by the pathophysiological processes that underlie VLOSLP. Within 4-5 years, more than 50% of VLOSLP patients suffer from dementia. Even in patients with VLOSLP without dementia, cognitive impairment is prevalent. As a result, it is controversial whether the risk of dementia is due to VLOSLP or a product of an early misdiagnosis.

DLB, in which visual hallucinations appear early and can present with hallucinations in other modalities, is a close differential to VLOSLP. Nonsystematized delusions, on the other hand, are seen in dementia. Prodromal presentation in DLB has persistent visual hallucinations and psychomotor retardation [5]. A decade prior, rapid eye movement (REM) sleep disorder, increased neuroleptic susceptibility, and evidence of Parkinsonism were some other features of DLB.

On a single-photon emission computerized tomography (SPECT) scan, the biomarker suggestive of DLB is the "cingulate island sign," which can be seen even in some cases of VLOSLP [1]. Vascular elements are linked to late-life psychosis in individuals with cognitive impairment, irrespective of any evidence of cerebrovascular lesions [6], and long-term hypertension correlates with abnormalities in white matter, specifically in its microstructure [7]. Both of these possibilities were ruled out. MRI of the brain findings of white matter hyperintensities in periventricular and subcortical frontoparietal regions are evidence of cognitive decline and VLOSLP. Late-life psychosis is closely associated with psychosocial aspects, such as retirement from a job and getting isolated socially, which are other important predisposing factors.

## Conclusions

In conclusion, VLOSLP's underlying neurological mechanisms are complex. The patient presenting with VLOSLP requires detailed physical examination, neurological assessment, laboratory investigations, and neuroimaging to rule out any underlying pathology. Hence, more systematic studies, including neuropathological and neuroimaging studies, are required to identify specific biomarkers that will explain the function of these neurobiological factors. These biomarkers will be categorized as per their causation. Furthermore, acquiring a particular biomarker would allow clinicians to diagnose VLOSLP more precisely, distinguish it from other overlapping clinical features of dementia, and provide the patient with effective treatment. We anticipate that it will encourage a phased care approach to the diagnosis of such cases in India, where long-term follow-ups and increasing population demands play a crucial role, which will ultimately be less invasive for the patient and will minimize society's costs.
